# Diet composition of an escaped captive‐born southern tamandua (*Tamandua tetradactyla*) in a nonnative habitat in Asia

**DOI:** 10.1002/ece3.9175

**Published:** 2022-08-04

**Authors:** Nick Ching‐Min Sun, Chung‐Chi Lin, Chun‐Chieh Liang, Hou‐Feng Li

**Affiliations:** ^1^ Department of Entomology National Chung Hsing University Taichung City Taiwan; ^2^ IUCN SSC Pangolin Specialist Group, Zoological Society of London London UK; ^3^ Department of Biology National Changhua University of Education Changhua City Taiwan

**Keywords:** anteater, exotic species, myrmecophagy, natural predatory instincts, *tamandua*, zoo

## Abstract

Studies on the role of natural predatory instincts in captive‐born mammalian myrmecophagy are rare. Consequently, researchers rely extensively on case reports to learn more about the contexts in which predatory behavior occurs among such animals. In this study, we recorded an uncommon case of a captive‐born southern tamandua (*Tamandua tetradactyla*) that accidentally escaped from a zoo into a nonnative habitat in Asia. The southern tamandua was found alive 3 months later. Two fresh fecal samples were obtained, and the diet composition was examined. Three termite species (one family, three genera), and 14 ant species (four subfamilies, nine genera) were identified in the fecal samples. The studied southern tamandua preyed on terrestrial and arboreal ants and termites, as the wild populations of its species do. Ants of the subfamily Myrmicinae and termites of the subfamily Nasutitermitinae were the most abundant prey items in the samples, which is consistent with related reports on the wild populations. Soldier ants constituted <1% of the prey items in the fecal samples, suggesting that the southern tamandua likely avoided preying on ants of the soldier caste. Fungus‐growing termites *Odontotermes* (Isoptera: Macrotermitinae), which are not native to neotropical regions, were also ingested by the southern tamandua. This study provides information on how a captive‐born mammalian myrmecophagy applies its natural feeding instincts in nonnative natural settings.

## Graphical abstract

Captive‐born lesser anteater can survive in nonnative habitat by expanding their food acceptance. The escaped lesser anteater fed on both sympatric and nonsympatric ant and termite taxa in Taiwan and survived for 3 months.

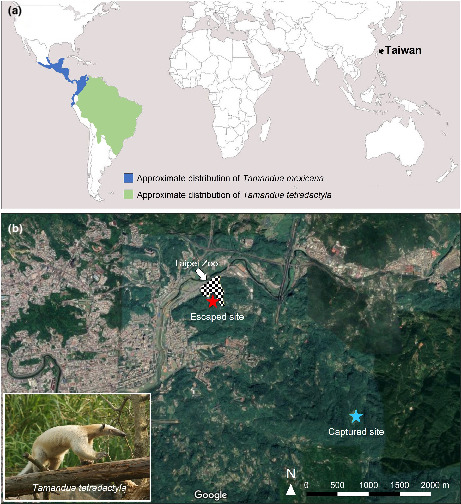

## 1 INTRODUCTION

**FIGURE 1:**
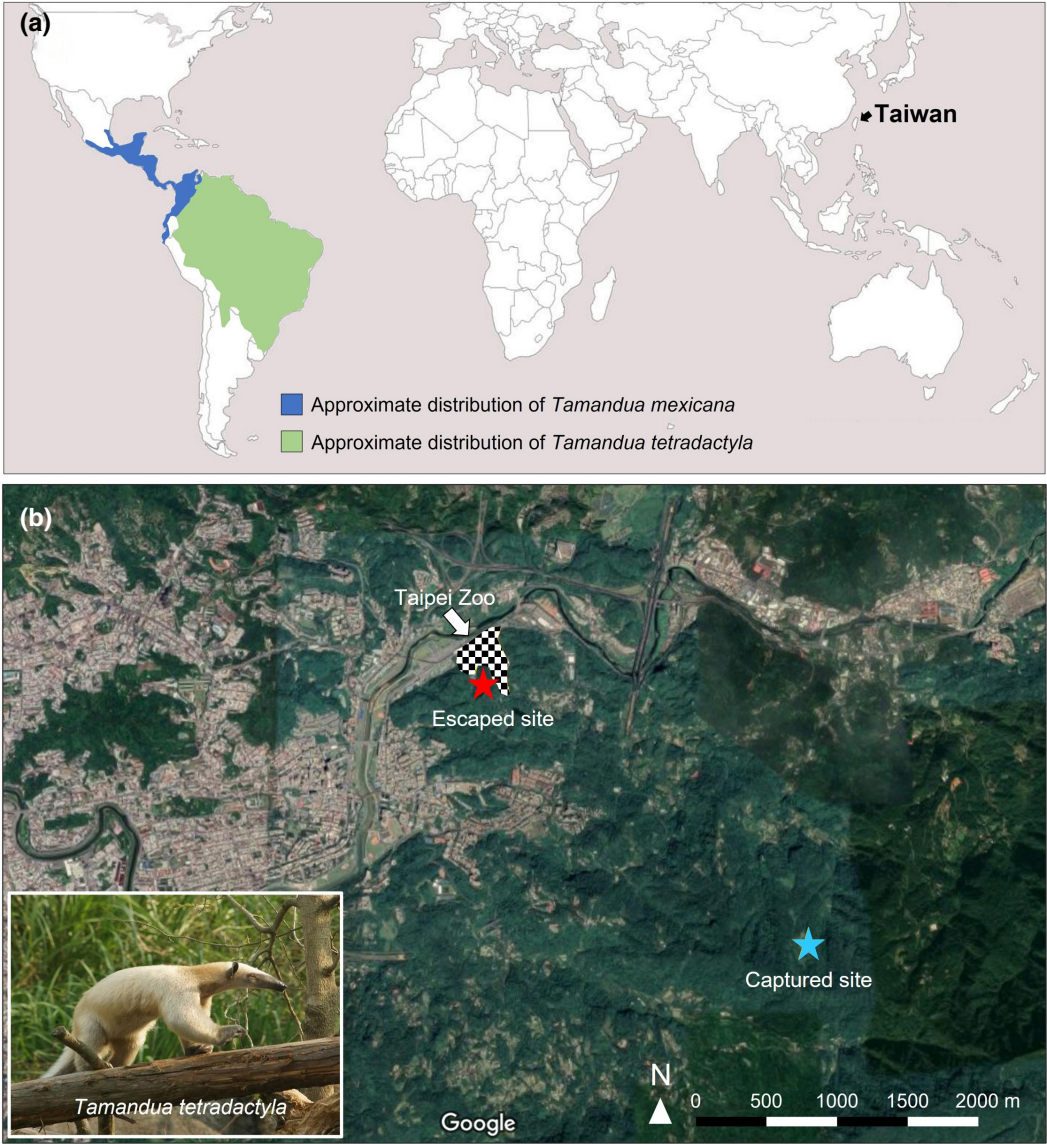
Geographical distribution of *tamandua* spp. and the study site, Taipei, Taiwan (a). Map modified from Villa and Cervantes (2003); Hall (1981); Wetzel (1985); and Hayssen (2011). Locations where the *T*. *tetradactyla* escaped and was captured (b). Tamandua photo credit: Yi‐tang Chang.

Xenarthra is one of the four main clades of placental mammals, which includes armadillos, sloths, and anteaters (Gardner, 2008). Anteaters are the least diverse xenarthran group and include pygmy anteaters *Cyclopes didactylus*, giant anteaters *Myrmecophaga tridactyla*, and tamanduas *Tamandua* spp. (Gibb et al., 2016). Two species of tamandua have been described: *T*. *mexicana*, also called the northern tamandua, which is distributed from southern Mexico to the northwest Andes in South America (Navarrete & Ortega, 2011), and *T*. *tetradactyla*, also called the southern tamandua, which lives in northern and central South America east of the Andes (Hayssen, 2011; Figure 1a). Tamanduas occur in tropical and subtropical areas and inhabit diverse habitats, including evergreen forests, transitional forests, savanna, and areas degraded by agricultural use (Hayssen, 2011; Montgomery, 1985; Navarrete & Ortega, 2011; Rodrigues et al., 2001). Both species are capable of moving, feeding, and resting on the ground and in trees (Handley, 1976; Lubin & Montgomery, 1981; Montgomery, 1985). Tamanduas are highly specialized predators, feeding predominantly on ants and termites (Lubin & Montgomery, 1981; Montgomery, 1985; Vaz et al., 2012) and other small arthropods (Hayssen, 2011; Redford, 1985), along with occasional fruit (Meritt, 1976). The proportions of prey items consumed by tamanduas vary among species, individuals, and regions (Montgomery, 1985; Pages, 1970; Rodrigues et al., 2008). Seasonal variations in prey composition and nutritional content among wild tamanduas have also been reported (Lubin & Montgomery, 1981). Consequently, meeting the dietary and behavioral needs of captive and captive‐born tamanduas, such as those bred in modern zoos, is challenging (Oyarzun et al., 1996). Captive individuals typically receive artificial or commercial feed (Catapani et al., 2018).

The abundance and ready availability of food supplies in captivity may affect animals' natural predatory instincts (Maple, 1980; Poole, 1987). The factors that affect the survival of captive‐born or reintroduced animals in the wild include prerelease training, habitat characteristics, food supplies, and anthropogenic disturbances in the environment (Maran et al., 2009). Jiménez Pérez et al. (2015) reported the survival rate of reintroduced giant anteaters in Iberá Nature Reserve, Argentina was 76% (11 died out of 47 released animals). There have been few studies on the role of natural predatory instincts in captive‐born mammalian myrmecophagy in the wild. Here, we report a rare case of a 3‐year‐old captive‐born female southern tamandua that accidentally escaped from Taipei Zoo into a nonnative habitat. The southern tamandua had not previously lived in the wild nor fed on natural prey items. The studied southern tamandua was found alive after 3 months in the wild. After locating the southern tamandua, we collected two fresh fecal samples and investigated the southern tamandua's diet composition. Specialist species are more susceptible to nonnatives diet than are generalist species because of their narrow feeding habits. Therefore, how this captive‐born southern tamandua applied its natural feeding instincts in the wild and how it selected nonnative prey are of interest. We conducted this study to analyze what prey items the captive‐born southern tamandua consumed in the nonnative habitat and whether these prey items were similar to those consumed by tamanduas in native habitats. Given that this is the first report of a captive‐born southern tamandua surviving for 3 months in a nonnative habitat, it provides valuable insights regarding the adaptation of exotic species and the detailed dietary composition of the southern tamandua, further elucidating the characteristics and behaviors of this highly specialized predator.

## 2 METHODS

### 2.1 Study area

The study area is close to Taipei Zoo (24.994676, 121.586293) in northern Taiwan (Figure 1b). Taipei Zoo is bordered by hills to the south and east and has an average elevation of 100 m. The climate of northern Taiwan is generally subtropical. The average monthly temperature in 2020 was 22.6°C (range: 16.2°C in January to 28.3°C in July; ShengKeng station, Taiwan Central Weather Bureau, http://www.cwb.gov.tw). The annual precipitation in 2020 was 2132.5 mm (Taiwan Central Weather Bureau). The vegetation cover of the study area consists of evergreen broad‐leaved forests, bamboo forests, grasslands, and farmlands.

### 2.2 Subject

The southern tamandua was captive‐born and brought to Taipei Zoo at 12 months in August 2018. The captive environment at the zoo had natural structural features, including tree trunks, branches, and plants with dirt substrates. The southern tamandua received 300 g of feed, composed of meal worms, bee pupae, fruits, chitin powder, and vegetables, daily. As of November 3, 2019, the southern tamandua weighed 6.2 kg. On November 28, 2019, the southern tamandua was paired. On May 10, 2020, the southern tamandua gave birth. The cub was nursed by the studied southern tamandua. At 2:30 a.m. on September 1, 2020, the studied southern tamandua escaped from Taipei Zoo with the cub and reached a nearby forest (Figure 1b). The cub was found alive the next day in the culvert of the zoo. After 97 days, on December 6, 2020, the studied southern tamandua was found in a tree den in a secondary forest 3 km away from the zoo (Figure 1b), and its identity was confirmed through microchip scanning. The southern tamandua was subsequently brought to Taipei Zoo for clinical examination. Its body weight was 5.5 kg at the time. On the same day, the southern tamandua received 450 g of regular feed.

### 2.3 Analysis of fecal composition

**FIGURE 2:**
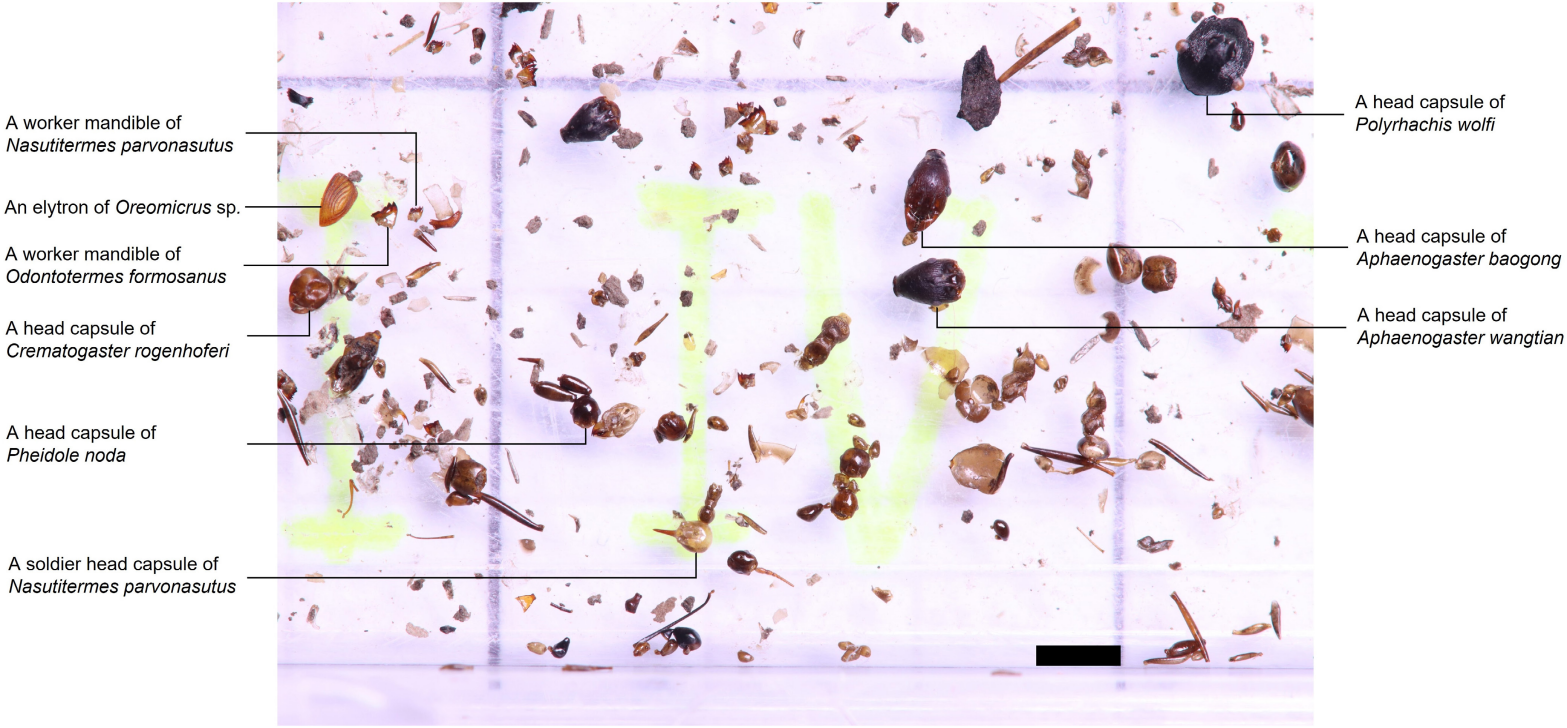
Macrophotograph of cuticular remains of prey in southern tamandua (*Tamandua tetradactyla*) feces. The feces were collected from a captive‐born southern tamandua that escaped from Taipei zoo and lived in the wild for 3 months in Taiwan. Scale bar = 2 mm.

On December 7, two of the southern tamandua's fecal samples were collected from its enclosure. The feces from which the samples were collected were defecated 30 min apart. In captivity, tamanduas normally defecate every 2 to 6 days (Meritt, 1975), indicating the southern tamandua feces collected in this study presumably represented its diet composition in the wild from within the preceding week. Both of the fecal samples consisted of the cuticular remains of ants and termites. We sampled 1 g of dry mass from each defecation for prey composition analysis. We performed fecal content analysis and prey item identification to extract prey items and debris by following the fecal filtering procedures proposed by Sun et al. (2019). The filtered prey items were macrophotographed and digitized (see Sun et al., 2019 for details). The termites and ants were identified to species level, and the number of individuals was counted using the macrophotographs (Sun et al., 2019). The morphological characteristics of ant head capsules were used for species identification and individual quantification. In termites, the left mandible is more morphologically diverse than the right mandible, and the numbers of left and right mandibles in the feces of animals consuming termites do not differ significantly (Liang, 2017). We therefore used termite left mandibles for species identification and individual quantification. For conehead soldier termites of genus *Nasutitermes*, head capsules were used for species identification and individual quantification (as illustrated in Figure 2). Finally, the prey item information collected in this study (such as subfamily, genus, and lifestyle) was compared with that reported in previously published literature.

## 3 RESULTS

A total of 1019 termite individuals across three species (one family, three genera) and 583 ant individuals across 14 species (four subfamilies, nine genera) were identified from the fecal samples (Table 1). Two genera of termites, (*Reticulitermes* and *Odontotermes*) and six genera of ants (*Technomyrmex*, *Polyrhachis*, *Aphaenogaste*, *Strumigenys*, *Tetramorium*, and *Brachyponera*) were the first recorded prey items. The ant subfamily Myrmicinae appeared in the samples in the highest frequency (eight species, 57.1% of the total ant species consumed) and number (534 individuals, 92.1% of the total ant individuals consumed) than other ant subfamilies. The arboreal ant *Crematogaster rogenhoferi* comprised more than half of the ant individuals consumed (333 individuals, 63.4%). Moreover, soldier ants comprised an extremely low percentage of the ant individuals consumed (three individuals, <1%). Termites of the genus *Nasutitermes* comprised the majority of the termite individuals consumed (748 individuals, 73.4%). Among the *Nasutitermes* consumed, 629 and 119 were workers and soldiers, respectively. *Odontotermes* was the second most common genus of termites in the samples, comprising 24.9% of the termite individuals consumed. All of the *Odontotermes* workers consumed were major workers. Six elytra of a termitophilous beetle, *Oreomicrus* sp., were also present in the fecal samples (Figure 2).

**TABLE 1:** Review of *Tamandua* spp*.* diet composition compared with that of the southern tamandua from the present study. The first two columns of number of prey species consumed are from previous studies.

Prey items	No. of prey species consumed				Lifestyles
	*T*. *mexicana*	*T*. *tetradactyla*	Present study (worker, soldier)		
Ant					
Dolichoderinae					
*Azteca*	?^h^	1^c,d^			Arboreal
*Monacis*		1^d^			Arboreal
*Technomyrmex*			1	(8, NA)	Terrestrial/arboreal
Dorylinae					
Army ant (Unidentified)		1^e^			Terrestrial
*Neivamyrmex*	1^h^				Terrestrial
Ectatomminae					
*Ectatomma*	2^h^				Terrestrial
*Gnamptogenys*		1^a^			Terrestrial/arboreal
Ecitoninae					
*Eciton*	1^h^				Terrestrial
Formicinae					
*Brachymyrmex*	2^h^	1^a^			Terrestrial
*Camponotus*	5^h^	6^a,d^	1	(3, 0)	Terrestrial/arboreal
*Nylanderia*		1^a^			Terrestrial
*Polyrhachis*			3	(6, NA)	Terrestrial/arboreal
Myrmicinae					
*Acromyrmex*	1^h^	1^a^			Terrestrial/arboreal
*Aphaenogaste*			2	(68, NA)	Terrestrial/arboreal
*Cephalotes*		2^h^			Arboreal
*Crematogaster*	2^h^	2^a^	1	(333, NA)	Arboreal
*Monomorium*	1^h^				Terrestrial/arboreal
*Pheidole*	2^h^	4^a^	3	(123, 3)	Terrestrial/arboreal
*Solenopsis*	5^h^	2^a^			Terrestrial
*Strumigenys*			1	(1, NA)	Terrestrial
*Tetramorium*			1	(9, NA)	Terrestrial/arboreal
*Trachymyrmex*		1^a^			Terrestrial
Ponerinae					
*Brachyponera*			1	(29, NA)	Terrestrial
*Heteroponera*		1^a^			Terrestrial/arboreal
*Odontomachus*	1^h^				Terrestrial
*Pachycondyla*		1^a^			Terrestrial
Termite					
Kalotermitidae					
*Calcaritermes*	?^f^				Terrestrial
Macrotermitinae					
*Odontotermes*			1	(232*, 22)	Subterranean
Nasutitermitinae					
*Nasutitermes*	?^f^	4^c,d,g^	1	(629, 119)	Terrestrial/arboreal
Rhinotermitidae					
*Coptotermes*	?^f^				Terrestrial
*Reticulitermes*	?^f^		1	(15, 2)	Terrestrial
Termitinae					
*Armitermes*	?^f^				Arboreal
*Microcerotermes*	?^f^	1^c^			Terrestrial/arboreal
Beetle					
Hydrophilidae					
*Oreomicrus*			1^b^		Termitophilous

*Note*: * = major workers.

References used: [a] Gallo et al., 2017; [b] Liang & Li, 2016; [c] Lubin et al., 1977; [d] Lubin & Montgomery, 1981; [e] Montgomery & Lubin, 1977; [f] Navarrete & Ortega, 2011; [g] Oyarzun et al., 1996; [h] Sandoval‐Gómez et al., 2012.

## 4 DISCUSSION

Although the findings of this study are based on limited fecal samples, we were able to collect detailed information regarding the prey composition of the southern tamandua. Our findings also provide insights into the feeding adaptation of a captive‐born southern tamandua in a nonnative habitat. Overall, the prey composition of the studied southern tamandua exhibited some common patterns with those of wild populations of tamanduas in South America. For example, the studied southern tamandua consumed both terrestrial and arboreal ants and termites, which is consistent with previous records (Table 1). The ant subfamilies Myrmicinae and Formicinae exhibit the highest species richness in the diets of wild tamanduas (Table 1). However, in this study, high species richness was only observed for the subfamily Myrmicinae. This was likely due to the limited number of fecal samples collected or the availability of Myrmicinae is generally abundant in suburban Taiwan. In addition, soldier ants comprised <1% of the ant individuals present in the fecal samples, which suggests that the southern tamandua likely avoided preying on colonies with large numbers of ants of the soldier caste. Social insects typically have well‐developed chemical and physical defenses (Redford, 1985), and the defense behavior of ants and termites is effective in repelling anteaters (Montgomery & Lubin, 1977).

Montomery (Montgomery, 1985) and Lubin and Montgomery (1981) reported that *Nasutitermes* termites are an important part of the diets of both tamandua species. *Nasutitermes parvonasutus* are commonly found in suburban Taiwan (Liang & Li, 2016) and were the species of which the southern tamandua in this study consumed the most. Although *N*. *parvonasutus* do not inhabit neotropical regions, *Nasutitermes* spp. are found commonly in south America (Constantino, 2020). We speculated the studied southern tamandua preyed on a significant amount of *N*. *parvonasutus* likely due to the display of their natural predatory instincts. *N*. *parvonasutus* does not build arboreal nests but build shelter tubes on tree trunks; mature colonies of this species may also build nests underground (Liang & Li, 2016). In addition, *Nasutitermes* soldiers are present in greater numbers in nests and in lower numbers in shelter tubes and decomposing wood; and the defense of soldiers is effective in repelling large vertebrate predators, such as tamanduas (Lubin & Montgomery, 1981). Remarkably, a termitophilous beetle, *Oreomicrus* sp. (Hydrophilidae: Sphaeridiinae: Omicrini), was identified in the southern tamandua's fecal samples. *Oreomicrus* sp. was observed in *N*. *parvonasutus* tunnels in decomposing wood by Liang and Li (2016) in Taiwan. The southern tamandua likely ingested the termitophilous beetles together with *N. parvonasutus* in shelter tubes and decomposing wood during foraging. Montgomery (1985) also reported that tamanduas ingest certain commensal arthropods of social insects.

The fungus‐growing termite *O*. *formosanus* does not occur in neotropical regions, whereas in Taiwan, it is a widespread species (Chiu et al., 2010). *O*. *formosanus* constructs subterranean nests and gallery systems and builds foraging shelter tubes on the surfaces of tree trunks, dead grass, tree branches, leaf litter, and other natural materials (Chiu et al., 2018). The studied southern tamandua ingested a considerable amount of *O*. *formosanus*, which was probably because of prey accessibility and availability in the environment (Gallo et al., 2017; Reiss, 2000). In addition, only major worker of *O*. *formosanus* was present in the fecal samples; no minor workers, alates, or nymphs were present. Generally, major worker termites are responsible for foraging and building shelter tubes on the ground, whereas minor workers are more commonly involved in feeding and nursing within underground nest chambers (de Oliveira et al., 2021; Wang et al., 2009). Hence, we speculated that the southern tamandua fed on *O*. *formosanus* foraging aboveground instead of excavating underground nest chambers.

*Reticulitermes* are also not native to South America, and they were recently introduced to Chile, Argentina, and Uruguay (Austin et al., 2005; Constantino, 2002). The distribution of the introduced *Reticulitermes* does not overlap with that of south tamandua. *R*. *flaviceps* is an endemic species and only found in Taiwan, and they are commonly found in lowland forest and also build foraging tubes on the ground (Wu et al., 2019). Hence, we believe the studied tamandua preyed on *R*. *flaviceps* likely due to the availability on the ground.

In Brazil, Rodrigues et al. (2001) reported that translocated southern tamandua usually remain close to their release locations, with the largest observed distance between the release site and the recapture site being 2.17 km. This indicates that southern tamandua may adapt to new environments rapidly. In this study, the distance from Taipei Zoo to where the studied southern tamandua was located was long—approximately 3 km. This was likely related to anthropogenic disturbances in the environment, such as free‐roaming dogs (i.e., dogs that have owners but are not restricted to a prescribed indoor or outdoor space). The density of free‐roaming dogs in northern Taiwan is high and negatively affects the wildlife these dogs encounter (Yen et al., 2019).

In summary, this study reveals the adaptations of a captive‐born animal in a nonnative habitat and provides insights into how captive‐born animals apply their natural feeding instincts in natural settings. To expand upon the current results and further elucidate the dietary characteristics of tamanduas in natural and captive settings, the gut microbiomes of wild and captive southern tamandua were analyzed; the results will be provided in a future article.

## AUTHOR CONTRIBUTIONS

**Nick Ching‐Min Sun:** Conceptualization (lead); data curation (equal); formal analysis (equal); investigation (lead); methodology (equal); validation (equal); visualization (equal); writing – original draft (lead); writing – review and editing (supporting). **Chung‐Chi Lin:** Conceptualization (supporting); data curation (equal); formal analysis (equal); investigation (supporting); methodology (supporting); supervision (supporting); writing – original draft (supporting). **Chun‐Chieh Liang:** Data curation (supporting); formal analysis (supporting); methodology (equal); supervision (supporting); writing – original draft (supporting). **Hou‐Feng Li:** Conceptualization (equal); data curation (equal); formal analysis (equal); funding acquisition (lead); investigation (supporting); methodology (equal); project administration (equal); supervision (lead); writing – original draft (supporting); writing – review and editing (equal).

## CONFLICT OF INTEREST

None declared.

## Data Availability

All the data are presented in the article (Table 1). No additional data will be uploaded at other place.
